# The Japanese Catheter Ablation Registry (J‐AB): Annual Report in 2023

**DOI:** 10.1002/joa3.70173

**Published:** 2025-08-21

**Authors:** Kengo Kusano, Koichi Inoue, Koshiro Kanaoka, Koji Miyamoto, Yasuo Okumura, Yu‐Ki Iwasaki, Kazuhiro Satomi, Seiji Takatsuki, Kohki Nakamura, Seigo Yamashita, Masaharu Masuda, Yoshitaka Iwanaga, Shoko Chishaki‐Kawabata, Teiichi Yamane, Wataru Shimizu, Hiroshi Tada, J‐AB registry investigators

**Affiliations:** ^1^ Department of Cardiovascular Medicine National Cerebral and Cardiovascular Center Suita Japan; ^2^ Cardiovascular Division National Hospital Organization Osaka National Hospital Osaka Japan; ^3^ Department of Medical and Health Information Management National Cerebral and Cardiovascular Center Suita Japan; ^4^ Division of Cardiology, Department of Medicine Nihon University School of Medicine Tokyo Japan; ^5^ Department of Cardiovascular Medicine Nippon Medical School Tokyo Japan; ^6^ Department of Cardiology Tokyo Medical School Tokyo Japan; ^7^ Department of Cardiology Keio University School of Medicine Tokyo Japan; ^8^ Division of Cardiology Gunma Prefectural Cardiovascular Center Maebashi Japan; ^9^ Division of Cardiology, Department of Internal Medicine The Jikei University Katsushika Medical Center Tokyo Japan; ^10^ Cardiovascular Center Kansai Rosai Hospital Amagasaki Japan; ^11^ Department of Cardiology Sakurabashi‐Watanabe Hospital Osaka Japan; ^12^ Division of Cardiology Department of Internal Medicine The Jikei University School of Medicine Tokyo Japan; ^13^ Department of Cardiovascular Medicine New Tokyo Hospital Matsudo Japan; ^14^ Department of Cardiovascular Medicine, Faculty of Medical Sciences University of Fukui Fukui Japan

**Keywords:** catheter ablation, complication, J‐AB, nationwide registry

## Abstract

The Japanese Catheter Ablation (J‐AB) registry, started in August 2017, is a voluntary, nationwide, multicenter, prospective, observational registry performed by the Japanese Heart Rhythm Society (JHRS) in collaboration with the National Cerebral and Cardiovascular Center. From January 2022, the data registration system was changed from the Research Electronic Data Capture (REDCap) system to the Fountayn system. The purpose of this registry is to collect the details of target arrhythmias, the ablation procedures, including the type of target arrhythmias, outcomes, and acute complications in real‐world settings. During the year of 2023, we have collected a total of 102 584 procedures (mean age of 66.9 years and 65.2% male) from 549 participant hospitals. Detailed data were shown in figures and tables.

## Graphical abstract

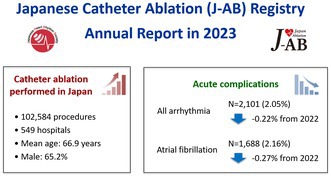

Catheter ablation has become an established therapy for the management of various cardiac arrhythmias, and the procedure number has been dramatically increasing. However, little is known about the details of target arrhythmias, the ablation procedures, including the type of target arrhythmias, outcomes, and acute complications in real‐world settings.

There are several preceding registries of catheter ablation, but the majority of which collected data from selected centers and/or selected arrhythmia and/or specified months to reveal the current status of ablations. Accordingly, we conducted a nationwide, multicenter, prospective, observational registry in Japan, named the Japanese Catheter Ablation (J‐AB) registry, aiming to register all catheter ablation cases in Japan [1]. This registry has been performed by the Japanese Heart Rhythm Society (JHRS) in collaboration with the National Cerebral and Cardiovascular Center using initially the Research Electronic Data Capture (REDCap) system. From January 2022, the data registration system was changed from REDCap to the Fountayn system, renamed J‐AB 2022, and the research protocol was approved by the central ethics review board of the JHRS (No. 2021001, approved at December 16, 2021), and participation is permitted with the approval of the director of each data‐providing institution. All participants were provided informed consent either by a written paper or by an opt‐out fashion and could withdraw their consent at any time. This study was also registered in the UMIN Clinical Trial Registry (UMIN 000028288) and ClinicalTrials.gov (NCT03729232). This J‐AB registry started in August 2017; since then, the number of participating hospitals has increased to over 500 at the end of 2022. Annual data during the years of 2018 to 2022 has been already reported [2, 3, 4, 5, 6], and now we report here the annual report of the results during the year of 2023. Figure 1 showed that the cumulative procedures during the year of 2023. Figure 2 showed that the number and rate of the target arrhythmias. AF ablation was the leading procedure (75.0% of all ablation procedures) in 2023, and the percentage of patients over 75 years of age was 33.1% in 2023. Patient characteristics, acute outcomes, and acute complications of all and AF procedures were shown in Tables 1, 2, 3, respectively.

**FIGURE 1:**
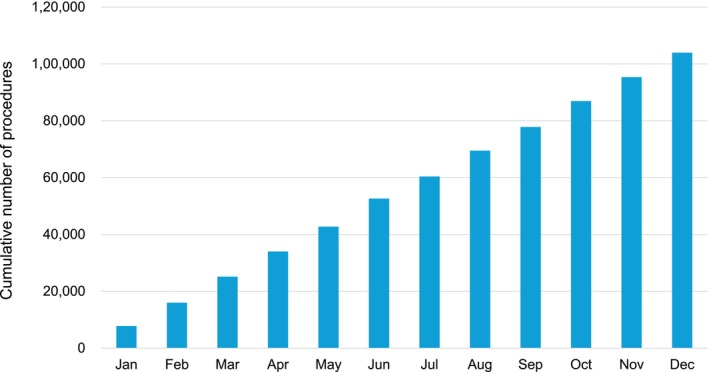
Cumulative number of registered hospitals (red line) and the patients (blue bars) during the year of 2022.

**FIGURE 2:**
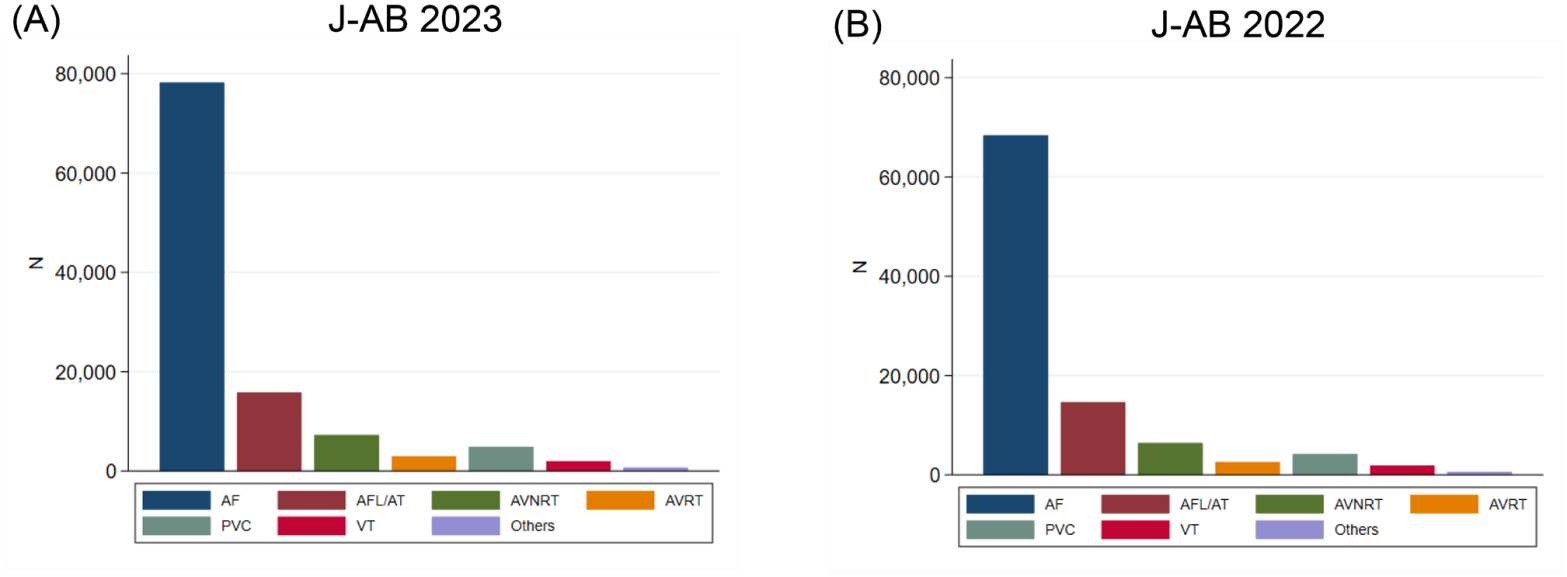
The number and rate of the target arrhythmias in the J‐AB 2022 (90 042 procedures; A) and 2021 (89 609 procedures; B). AF, atrial fibrillation; AFL, atrial flutter; AT, atrial tachycardia; AVNRT, atrioventricular nodal reentrant tachycardia; AVRT, atrioventricular reentrant tachycardia; IVC, inferior vena cava; PVC, premature ventricular contraction; TV, tricuspid valve; VT, ventricular tachycardia.

**TABLE 1:** Patient characteristics.

	All procedures	Atrial fibrillation (AF)			Atrial flutter (AFL)/atrial tachycardia (AT)				Atrioventricular nodal reentrant tachycardia	Atrioventricular reentrant tachycardia	Premature ventricular contraction	Ventricular tachycardia (VT)		
		All AF	Paroxysmal AF (PAF)	Non‐PAF	All AFL/AT	IVC‐TV isthmus dependent AFL	Uncommon AFL macro AT	Focal AT				Idiopathic VT	VT due to ischemic cardiomyopathy	VT due to nonischemic cardiomyopathy
*N*	1,02,584	78,196	42,881	35,314	15,798	10,086	4039	3054	7254	2981	4849	792	525	523
Age, mean ± SD	66.9±13.1	68.6±10.7	68.5±11.1	68.7±10.2	69.5±12.3	69.5±11.7	71.5±11.2	67.2±14.9	59.4±16.7	48.2±20.7	58.6±16.5	54.9±19.5	69.1±9.6	64.5±12.6
Gender, male	66,876 (65.2)	52,894 (67.6)	27,194 (63.4)	25,700 (72.8)	10,563 (66.9)	7606 (75.4)	2343 (58.0)	1393 (45.6)	3015 (41.6)	1899 (63.7)	2703 (55.7)	531 (67.0)	487 (92.8)	436 (83.4)
*Heart diseases*														
IHD														
No	92,069 (89.7)	70,287 (89.9)	38,480 (89.7)	31,806 (90.1)	13,849 (87.7)	8748 (86.7)	3513 (87.0)	2793 (91.5)	6921 (95.4)	2863 (96.0)	4359 (89.9)	703 (88.8)	—	478 (91.4)
Yes	9175 (8.9)	6888 (8.8)	3828 (8.9)	3060 (8.7)	1799 (11.4)	1258 (12.5)	472 (11.7)	236 (7.7)	249 (3.4)	77 (2.6)	439 (9.1)	83 (10.5)	—	39 (7.5)
Unknown	1340 (1.3)	1021 (1.3)	573 (1.3)	448 (1.3)	150 (0.9)	80 (0.8)	54 (1.3)	25 (0.8)	84 (1.2)	41 (1.4)	51 (1.1)	6 (0.8)	—	6 (1.1)
Cardiomyopathy														
No	94,140 (91.8)	71,846 (91.9)	40,451 (94.3)	31,394 (88.9)	14,222 (90.0)	9099 (90.2)	3499 (86.6)	2832 (92.7)	7084 (97.7)	2902 (97.3)	4424 (91.2)	706 (89.1)	483 (92.0)	—
Yes	7130 (7.0)	5341 (6.8)	1867 (4.4)	3474 (9.8)	1442 (9.1)	917 (9.1)	481 (11.9)	206 (6.7)	98 (1.4)	46 (1.5)	370 (7.6)	71 (9.0)	28 (5.3)	—
Unknown	1314 (1.3)	1009 (1.3)	563 (1.3)	446 (1.3)	134 (0.8)	70 (0.7)	59 (1.5)	16 (0.5)	72 (1.0)	33 (1.1)	55 (1.1)	15 (1.9)	14 (2.7)	—
Valve disease														
No	94,630 (92.2)	72,115 (92.2)	40,259 (93.9)	31,856 (90.2)	13,827 (87.5)	8944 (88.7)	3214 (79.6)	2727 (89.3)	7039 (97.0)	2904 (97.4)	4621 (95.3)	757 (95.6)	467 (89.0)	444 (84.9)
Yes	6841 (6.7)	5201 (6.7)	2125 (5.0)	3075 (8.7)	1878 (11.9)	1097 (10.9)	784 (19.4)	316 (10.3)	150 (2.1)	47 (1.6)	188 (3.9)	32 (4.0)	49 (9.3)	74 (14.1)
Unknown	1113 (1.1)	880 (1.1)	497 (1.2)	383 (1.1)	93 (0.6)	45 (0.4)	41 (1.0)	11 (0.4)	65 (0.9)	30 (1.0)	40 (0.8)	3 (0.4)	9 (1.7)	5 (1.0)
CHD														
No	100,113 (97.6)	76,569 (97.9)	41,993 (97.9)	34,575 (97.9)	15,119 (95.7)	9704 (96.2)	3732 (92.4)	2939 (96.2)	7148 (98.5)	2912 (97.7)	4768 (98.3)	774 (97.7)	513 (97.7)	514 (98.3)
Yes	1,352 (1.3)	736 (0.9)	388 (0.9)	348 (1.0)	585 (3.7)	338 (3.4)	263 (6.5)	105 (3.4)	45 (0.6)	40 (1.3)	41 (0.8)	14 (1.8)	4 (0.8)	5 (1.0)
Unknown	1,119 (1.1)	891 (1.1)	500 (1.2)	391 (1.1)	94 (0.6)	44 (0.4)	44 (1.1)	10 (0.3)	61 (0.8)	29 (1.0)	40 (0.8)	4 (0.5)	8 (1.5)	4 (0.8)

Abbreviations: CHD, congenital heart disease; IHD, ischemic heart disease; SD, standard deviation.

**TABLE 2:** Acute outcomes.

2023	*n* (%)	2022	*n* (%)	2023–2022, % change
*Pulmonary vein isolation of atrial fibrillation*	*n* = 77,906	*Pulmonary vein isolation of atrial fibrillation*	*n* = 67,967	
Ablation system		Ablation system		
RF alone	56,119 (72.0%)	RF alone	49,416 (72.7%)	−0.7
Ballon alone (Cryo, hot, laser)	15,525 (19.9%)	Ballon alone (Cryo, hot, laser)	13,399 (19.7%)	+0.2
RF + Ballon combination	6104 (7.8%)	RF + Ballon combination	5083 (7.5%)	+0.3
Others	158 (0.2%)	Others	69 (0.1%)	+0.1
Patient with a first session (*n* = 55,170)	*n* = 62,856	Patient with a first session	*n* = 55,170	
Success	62,499 (99.4%)	Success	54,960 (99.6%)	−0.2
Unsuccess	357 (0.6%)	Unsuccess	210 (0.4%)	+0.2
Unknown or others	0 (0.0%)	Unknown	0 (0.0%)	0.0
Patient with second session	*n* = 12,106	Patient with second session	*n* = 10,325	
Success	8478 (70.0%)	Success	7517 (72.8%)	−2.8
Unsuccess	29 (0.2%)	Unsuccess	31 (0.3%)	−0.1
Already isolated	3599 (29.7%)	Already isolated	2777 (26.9%)	+2.8
Patient with ≥ third session	*n* = 2927	Patient with ≥ third session	*n* = 2445	
Success	1120 (38.3%)	Success	1137 (46.5%)	−8.2
Unsuccess	9 (0.3%)	Unsuccess	6 (0.2%)	+0.1
Already isolated	1798 (61.4%)	Already isolated	1302 (53.3%)	+8.1
IVC‐TV isthmus dependent atrial flutter	*n* = 10,086	IV‐TV isthmus dependent atrial flutter (*n* = 9605)	*n* = 9292	
Success	10,037 (99.5%)	Success	9223 (99.3%)	+0.2
Unsuccess	49 (0.5%)	Unsuccess	69 (0.7%)	−0.2
Uncommon atrial flutter/atrial tachycardia	*n* = 4039	Uncommon atrial flutter/atrial tachycardia (*n* = 3957)	*n* = 3884	
Complete success	3517 (87.1%)	Complete success	3322 (85.5%)	+1.6
Partial success	382 (9.5%)	Partial success	407 (10.5%)	−1.0
Unsuccess	103 (2.6%)	Unsuccess	123 (3.2%)	−0.6
Unknown or others	37 (0.9%)	Unknown	32 (0.8%)	+0.1
Focal atrial tachycardia	*n* = 3054	Focal atrial tachycardia (*n* = 2894)	*n* = 2797	
Complete success	2601 (85.2%)	Complete success	2373 (84.8%)	+0.4
Partial success	322 (10.5%)	Partial success	298 (10.7%)	−0.2
Unsuccess	89 (2.9%)	Unsuccess	89 (3.2%)	−0.3
Unknown or others	42 (1.4%)	Unknown	37 (1.3%)	+0.1
Atrioventricular nodal reentrant tachycardia by slow‐fast	*n* = 6248	Atrioventricular nodal reentrant tachycardia by slow‐fast (*n* = 5534)	*n* = 5499	
Complete success	6108 (97.8%)	Complete success	5377 (97.8%)	0.0
Partial success	108 (1.7%)	Partial success	77 (1.4%)	+0.3
Unsuccess	16 (0.3%)	Unsuccess	28 (0.5%)	−0.2
Unknown or others	16 (0.3%)	Unknown	17 (0.3%)	0.0
Atrioventricular nodal reentrant tachycardia by fast‐slow	*n* = 702	Atrioventricular nodal reentrant tachycardia by fast‐slow (*n* = 573)	*n* = 607	
Complete success	674 (96.0%)	Complete success	587 (96.7%)	−0.7
Partial success	19 (2.7%)	Partial success	12 (2.0%)	+0.7
Unsuccess	3 (0.4%)	Unsuccess	6 (1.0%)	−0.6
Unknown or others	6 (0.9%)	Unknown	2 (0.3%)	+0.6
Atrioventricular nodal reentrant tachycardia by slow‐slow	*n* = 476	Atrioventricular nodal reentrant tachycardia by slow‐slow (*n* = 356)	*n* = 426	
Complete success	448 (94.1%)	Complete success	402 (94.4%)	−0.3
Partial success	18 (3.8%)	Partial success	19 (4.5%)	−0.7
Unsuccess	5 (1.1%)	Unsuccess	3 (0.7%)	+0.4
Unknown or others	5 (1.1%)	Unknown	2 (0.5%)	+0.6
Atrioventricular reentrant tachycardia by kent	*n* = 2981	Atrioventricular reentrant tachycardia by kent (*n* = 2670)	*n* = 2584	
Complete success	2819 (96.5%)	Complete success	2461 (96.9%)	−0.4
Unsuccess	71 (2.4%)	Unsuccess	51 (2.0%)	+0.4
Unknown or others	31 (1.1%)	Unknown	28 (1.1%)	0.0
Premature ventricular contraction	*n* = 4849	Premature ventricular contraction (*n* = 4,314)	*n* = 4167	
Complete success	3763 (77.6%)	Complete success	3215 (77.2%)	+0.4
Partial success	837 (17.3%)	Partial success	718 (17.2%)	+0.1
Unsuccess	198 (4.1%)	Unsuccess	198 (4.8%)	−0.7
Unknown or others	51 (1.1%)	Unknown	36 (0.9%)	+0.2
Idiopathic ventricular tachycardia	*n* = 792	Idiopathic ventricular tachycardia (*n* = 778)	*n* = 776	
Complete success	643 (81.2%)	Complete success	598 (77.1%)	+4.1
Partial success	99 (12.5%)	Partial success	132 (17.0%)	−4.5
Unsuccess	32 (4.0%)	Unsuccess	27 (3.5%)	+0.5
Unknown or others	18 (2.3%)	Unknown	19 (2.4%)	−0.1
Ventricular tachycardia due to ischemic cardiomyopathy	*n* = 525	Ventricular tachycardia due to ischemic cardiomyopathy (*n* = 459)	*n* = 445	
Complete success	384 (73.1%)	Complete success	328 (73.7%)	−0.6
Partial success	110 (21.0%)	Partial success	91 (20.4%)	+0.6
Unsuccess	16 (3.0%)	Unsuccess	11 (2.5%)	+0.5
Unknown or others	15 (2.9%)	Unknown	15 (3.4%)	−0.5
Ventricular tachycardia due to nonischemic cariomyopathy	*n* = 523	Ventricular tachycardia due to nonischemic cariomyopathy (*n* = 570)	545	
Complete success	312 (59.7%)	Complete success	314 (57.6%)	+2.1
Partial success	165 (31.5%)	Partial success	183 (33.6%)	−2.1
Unsuccess	30 (5.7%)	Unsuccess	26 (4.8%)	+0.9
Unknown or others	16 (3.1%)	Unknown	22 (4.0%)	−0.9

Abbreviations: IVC, inferior vena cava; RF, radiofrequency ablation; TV, tricuspid valve.

**TABLE 3:** Acute complications.

*N*	2023		2022		2023–2022, % change	
	All patient	AF	All patient	AF	All patient	AF
	1,02,584	78,196	90,042	68,378		
Complications during hospitalization	2101 (2.05%)	1688 (2.16%)	2046 (2.27%)	1660 (2.43%)	−0.22%	−0.27%
Major bleeding (BARC ≧ 2)	784 (0.76%)	576 (0.74%)	772 (0.86%)	592 (0.87%)	−0.1%	−0.13%
Cardiac tamponade	483 (0.47%)	335 (0.43%)	496 (0.55%)	356 (0.52%)	−0.08%	−0.09%
Embolism	129 (0.13%)	106 (0.14%)	127 (0.14%)	106 (0.16%)	−0.01%	−0.02%
Phrenic nerve paralysis	326 (0.32%)	320 (0.41%)	293 (0.33%)	285 (0.42%)	−0.01%	−0.01%
Esophagus	110 (0.11%)	110 (0.14%)	119 (0.13%)	119 (0.17%)	−0.02%	−0.03%
Gastric hypomotility	93 (0.09%)	93 (0.12%)	114 (0.13%)	114 (0.17%)	−0.04%	−0.05%
Pericardities	78 (0.08%)	64 (0.08%)	63 (0.07%)	56 (0.08%)	0.01%	0.00%
Sick sinus syndrome	138 (0.13%)	113 (0.14%)	125 (0.14%)	103 (0.15%)	−0.01%	−0.01%
Atrioventricular block	66 (0.06%)	22 (0.03%)	74 (0.08%)	20 (0.03%)	−0.02%	0.00%
Death during hospitalization	121 (0.12%)	50 (0.06%)	118 (0.13%)	43 (0.06%)	−0.01%	0.00%
Cardiac death	66 (0.06%)	19 (0.02%)	69 (0.08%)	22 (0.03%)	−0.02%	−0.01%
Related to ablation therapy	7 (0.007%)	3 (0.004%)	2 (0.002%)	1 (0.001%)	0.01%	0.00%
Non cardiac death	55 (0.05%)	31 (0.04%)	49 (0.05%)	21 (0.03%)	0.00%	0.01%
Related to ablation therapy	3 (0.003%)	3 (0.004%)	2 (0.002%)	2 (0.003%)	0.00%	0.00%

## Ethics Statement

This study was approved by the central ethics review board of the Japanese Heart Rhythm Society (No. 2021001, approved at December 16, 2021).

## Conflicts of Interest

Kengo Kusano: Speaker honoraria from Daiichi Sankyo Company Ltd., and Medtronic Japan, and research grants from Medtronic Japan, Abbott, Boston Scientific Japan, Biotronik Japan, GE Precision Healthcare LLC, Johnson & Johnson KK, and JSR. Koichi Inoue: Speaker honoraria from Daiichi Sankyo Company Ltd., Bristol Myers Squibb, Bayer Yakuhin, Nippon Boehringer Ingelheim, Johnson & Johnson KK, Medtronic Japan, and Boston Scientific Japan. Koji Miyamoto received funding/grants from Medtronic, Biosense Webster, Abbott, and Boston Scientific, honoraria/speakers' bureaus from Medtronic, Biosense Webster, Abbott, and Boston Scientific, and consultancies from Medtronic, Abbott, and Boston Scientific outside the submitted work and is affiliated with a department endowed by Medtronic outside the submitted work. Yasuo Okumura has received research funding from Medtronic Japan Co. Ltd., MicroPort CRM Japan, and Bayer Healthcare; and has accepted remuneration from AstraZeneca K.K. and Johnson & Johnson K.K. He is affiliated with endowed departments supported by Abbott, Boston Scientific Japan K.K., Medtronic Japan Co. Ltd., Japan Lifeline Co. Ltd., and Biotronik Japan. Kazuhiro Satomi received research funding irrelevant to this study from Abbott, Boston Scientific Japan, Biotronik Japan, and lecture fees from Medtronic Japan, Japan Lifeline. Seiji Takatsuki belongs to the Advanced Cardiac Arrhythmia Therapeutics Endowed Research Course, which is supported by Medtronic Japan, Japan Lifeline, Boston Scientific Japan, Abbott Japan, and Biotronik Japan. He has received lecture fees from Medtronic Japan, Japan Lifeline, Daiichi Sankyo Company Ltd., Boston Scientific Japan, and Abbott. Masaharu Masuda received research funding irrelevant to this study from Johnson and Johnson and lecture fees from Medtronic Japan, Daiichi‐Sankyo, and Boston Scientific Japan. Teiichi Yamane: Speaker honoraria from Medtronic Japan and BEG company, and research grants from Japan Lifeline. Wataru Shimizu: Speaker honoraria from Daiichi Sankyo Company Ltd., Nippon Boehringer Ingelheim, Pfizer, Johnson & Johnson KK, Boston Scientific Japan, Japan Lifeline, Medtronic Japan, and Abbott. Dr. Hiroshi Tada received honoraria for lectures or speakers bureaus from Daiichi Sankyo Company Ltd.; Novartis Pharma K.K.; Medtronic Japan Co. Ltd.; BIOTRONIK Japan Inc.; Bristol Myers Squibb; Boston Scientific Japan K.K. He received research grants (Investigator‐initiated study unrelated to the manuscript topic) from Abbott Medical Japan LLC; Daiichi Sankyo Company Ltd.; Nippon Boehringer Ingelheim Co. Ltd.; Otsuka Pharmaceutical Co. Ltd.; Eli Lilly Japan K.K.; Marubun Tsusyo K.K. Koshiro Kanaoka, Yu‐Ki Iwasaki, Kohki Nakamura, Yoshitaka Iwanaga, Shoko Chishaki‐Kawabata declare no conflicts of interest.
